# Supplementary material for: Where Is the Way Forward for New Media Empowering Public Health? Development Strategy Options Based on SWOT-AHP Model
Source: Int J Environ Res Public Health. 2022 Oct 6;19(19):12813. doi: 10.3390/ijerph191912813 (PMC9566796; doi:10.3390/ijerph191912813)
Supplement: Supplementary file 1 [file ijerph-19-12813-s001.zip › ijerph-1905637-supplementary.pdf]

Supplementary Table S1.

Group	Sub-factors	Accept or Reject（√ OR ×）	Estimated intensity
Strengths（S）	S1		1-5 (The higher the absolute value of the score, the more important it is）
	S2		
	S3		
	S4		
Opportunities（O）	O1		
	O2		
	O3		
	O4		
Weakness（W）	W1		（-1）-（-5） (The higher the absolute value of the score, the more important it is）
	W2		
	W3		
Threats（T）	T1		
	T2		
	T3		

Supplementary Table S2. Using S group to explain.

	**Comparisons among subfactors within group S**							
	**Equal Importance**	**Moderate importance**	**Strong importance**	**Very strong or demonstrated importance**	**Extreme importance**	**Importance between the above levels**	**If sub-factor A is less important than sub-factor B, then Intensity of Importance of A is the inverse of Intensity of Importance of B**	
	1	3	5	7	9	2,4,6,8	1/2,1/3,1/4,1/5,1/6,1/7,1/8,1/9	
S1								S1
S								S

1	2
S<br>1	S<br>3
S<br>1	S<br>4
S<br>2	S<br>1
S<br>2	S<br>2
S<br>2	S<br>2
S<br>2	S<br>2
S<br>3	S<br>3
S<br>3	S<br>3
S<br>3	S<br>3
S<br>3	S<br>3
S<br>4	S<br>4
S<br>4	S<br>4
S<br>4	S<br>4
S<br>4	S<br>4
